# Isolation and Structural Elucidation of Antiproliferative Compounds of Lipidic Fractions from White Shrimp Muscle (*Litopenaeus vannamei*)

**DOI:** 10.3390/ijms151223555

**Published:** 2014-12-17

**Authors:** Carmen-María López-Saiz, Carlos Velázquez, Javier Hernández, Francisco-Javier Cinco-Moroyoqui, Maribel Plascencia-Jatomea, Maribel Robles-Sánchez, Lorena Machi-Lara, Armando Burgos-Hernández

**Affiliations:** 1 Departamento de Investigación y Posgrado en Alimentos, Universidad de Sonora, Apartado Postal 1658, Hermosillo, Sonora 83000, Mexico; E-Mails: k_rmelita@hotmail.com (C.-M.L.-S.); fcinco@guayacan.uson.mx (F.-J.C.-M.); mplascencia@guayacan.uson.mx (M.P.-J.); rsanchez@guayacan.uson.mx (M.R.-S.); 2 Departamento de Ciencias Químico-Biológicas, Universidad de Sonora, Apartado Postal 1685, Hermosillo, Sonora 83000, Mexico; E-Mail: velaz@guayacan.uson.mx; 3 Unidad de Servicios de Apoyo en Resolución Analítica, Universidad Veracruzana, Xico, Veracruz 91190, Mexico; E-Mail: javmartinez@uv.mx; 4 Departamento de Investigación en Polímeros y Materiales, Universidad de Sonora, Apartado Postal 1685, Hermosillo, Sonora 83000, Mexico; E-Mail: lmachi@polimeros.uson.mx

**Keywords:** shrimp, chemoprevention, antiproliferative activity

## Abstract

Shrimp is one of the most popular seafood items worldwide, and has been reported as a source of chemopreventive compounds. In this study, shrimp lipids were separated by solvent partition and further fractionated by semi-preparative RP-HPLC and finally by open column chromatography in order to obtain isolated antiproliferative compounds. Antiproliferative activity was assessed by inhibition of M12.C3.F6 murine cell growth using the MTT (3-(4,5-dimethyl-2-thiazolyl)-2,5-diphenyl-2-*H*-tetrazolium bromide) assay. The methanolic fraction showed the highest antiproliferative activity; this fraction was separated into 15 different sub-fractions (M1–M15). Fractions M8, M9, M10, M12, and M13 were antiproliferative at 100 µg/mL and they were further tested at lower concentrations. Fractions M12 and M13 exerted the highest growth inhibition with an IC_50_ of 19.5 ± 8.6 and 34.9 ± 7.3 µg/mL, respectively. Fraction M12 was further fractionated in three sub-fractions M12a, M12b, and M12c. Fraction M12a was identified as *di*-ethyl-hexyl-phthalate, fraction M12b as a triglyceride substituted by at least two fatty acids (predominantly oleic acid accompanied with eicosapentaenoic acid) and fraction M12c as another triglyceride substituted with eicosapentaenoic acid and saturated fatty acids. Bioactive triglyceride contained in M12c exerted the highest antiproliferative activity with an IC_50_ of 11.33 ± 5.6 µg/mL. Biological activity in shrimp had been previously attributed to astaxanthin; this study demonstrated that polyunsaturated fatty acids are the main compounds responsible for antiproliferative activity.

## 1. Introduction

Naturally occurring bioactive extracts may benefit human health through inhibition of carcinogenic processes and cell death mechanisms [1,2], and compounds with these properties are termed chemopreventives [3]. Chemoprevention was originally defined by Sporn (1976) as the use of natural, synthetic, or biological chemical agents to reverse, suppress, or prevent cancer [4], which is the leading and second cause of death in economically developed and developing countries, respectively [5].

Shrimp’s muscle is rich in high quality proteins and low in fat content [6,7]; however, there is evidence that the lipidic fraction may exhibit chemopreventive and chemoprotective activities, including antiproliferative compounds which are capable of interfering in the cell cycle preventing uncontrolled cancer cell division.

The lipidic fraction of shrimp muscle, which is composed of carotenoids, phospholipids, neutral lipids (including cholesterol, triglycerides, free fatty acids, diglycerides, and monoglycerides) and glycolipids, represents 1%–2% (dry weight) [8] of the total weight. Several carotenoids (such as β-carotene, lycopene, and lutein) show different abilities in controlling the cell cycle [9], such as apoptosis [10,11,12,13] and inhibition of the cell cycle [10,14,15]. Polyunsaturated fatty acids (PUFAs) can also intervene in the cell cycle. According to Larsson (2004) [16], one of the mechanisms of PUFAs is through the modification of gene expression and signal transduction involved in the cell cycle [17]. Their functions underlie a multitude of cellular and physiological processes by directly modulating target gene expression and indirectly modulating other transcription factors [18]. However based on the above, the aim of the present study is to isolate and identify the compounds responsible for the antiproliferative activity that has been previously reported in shrimp muscle.

## 2. Results and Discussion

### 2.1. Lipidic Extraction and Partition

The chloroformic extraction of shrimp muscle had a yield of 1.860% ± 0.004% (dry basis). The lipidic fraction of shrimp muscle usually represents 1%–2% [8] of weight (dry basis) and consists of carotenoids [19], phospholipids [20], neutral lipids (including cholesterol, free fatty acids, mono, di and tryglicerides [21]), and glycolipids [22].

The chloroformic extract was partitioned in methanol-hexane; the yield for the methanolic fraction was 58% while the hexanic fraction was 42%. Both fractions were tested for antiproliferative activity.

#### Antiproliferative Activity of Fractions Obtained by Partition

Antiproliferative activity was measured by the MTT (3-(4,5-dimethyl-2-thiazolyl)-2,5-diphenyl-2-*H*-tetrazolium bromide) assay. In order to select the fraction with the highest antiproliferative activity, methanolic and hexanic fractions were tested in M12.C3.F6 murine cell lines at three different concentrations (Table 1).

**Table 1:** Percentage of proliferation of M12.C3.F6 murine exposed to methanolic and hexanic fractions at different concentrations.

Fraction	400 µg/mL	200 µg/mL	100 µg/mL
**Hexanic**	80.69 ± 4.62 ^a^	86.78 ± 10.13 ^a^	96.59 ± 6.94 ^a^
**Methanolic**	23.29 ± 5.61 ^b^	33.61 ± 13.8 ^b^	90.44 ± 8.27 ^a^

The results are represented as percentage of proliferation; the results shown are representative from three independent experiments. Different letters in a column represent significant differences (*p* < 0.05). Control cell cultures were incubated with DMSO (0.5%).

The hexanic fraction only inhibits M12.C3.F6 growth at a very low percentage while the methanolic fraction exerted the highest activity at the lower concentration (200 µg/mL). Even though the used concentration is still high, we had to consider that this fraction is composed of a large variety of compounds, where at least one of them is responsible for the biological activity. The methanolic fraction was selected for further fractioning.

### 2.2. Analysis of Lipidic Composition by RP-HPLC

**Figure 1:**
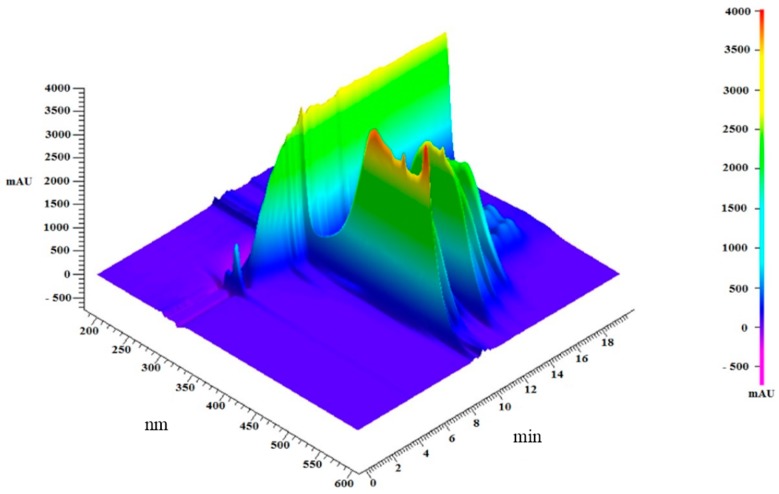
Spectrophotometric scanning of the lipidic extract from shrimp after reversed phase chromatography.

**Figure 2:**
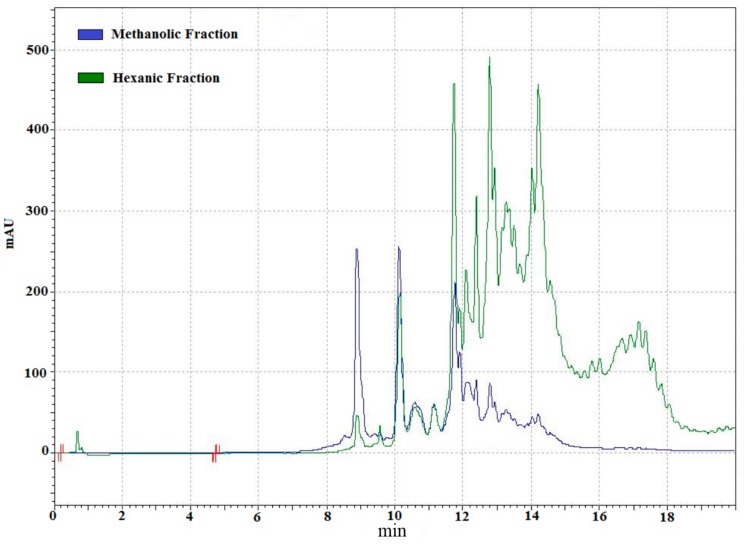
Chromatogram (450 nm) of methanolic and hexanic fractions obtained from lipidic extracts from shrimp.

In order to establish the absorbance at which the analysis was going to be carried out, the lipidic fraction was scanned from 190 to 600 nm (Figure 1). Signals were detected at various wavelengths; the signal at 275 nm is due to acetone in mobile phase; the highest signals were at 450 nm, these signals might be attributed to carotenoids in shrimp muscle [23]. Methanolic and hexanic fractions were analyzed to study their composition by RP-HPLC (Figure 2) and both were complementary. The more polar compounds were partitioned to the methanolic fraction while the non-polar remained in the hexanic phase. Several carotenoid compounds have been identified on shrimp, including astaxanthin [23], and astaxanthin esters in lower amounts [19,24], as well as β-criptoxanthin, α-carotene, β-carotene [25], canthaxanthin, lutein, zeaxanthin, and crustacyanin [26], which can also be found in this organism. Even though the highest signals were detected at 450 nm, other signals in the near UV spectrum were observed.

#### Antiproliferative Activity of Methanolic Sub-Fractions Obtained by Semi-Preparative RP-HPLC

Due to its higher antiproliferative activity, the methanolic fraction from white shrimp lipidic extract was further fractionated by RP-HPLC into 15 different sub-fractions (fractions were obtained every 1.33 min). In order to screen for the compounds with the highest antiproliferative activity, the contents from these fractions were tested against M12.C3.F6 cells at concentrations of 200 and 100 µg/mL (Table 2). Five (M8, M9, M10, M12, and M13) of the tested sub-fractions were selected for further analysis since all showed antiproliferative activity at 100 µg/mL without a significant difference among them. Fractions M8, M9, and M10 showed an orange color while M12 and M13 were colorless; this suggest that the nature of the compounds contained in these two groups of fractions significantly differ from each other.

**Figure 3:**
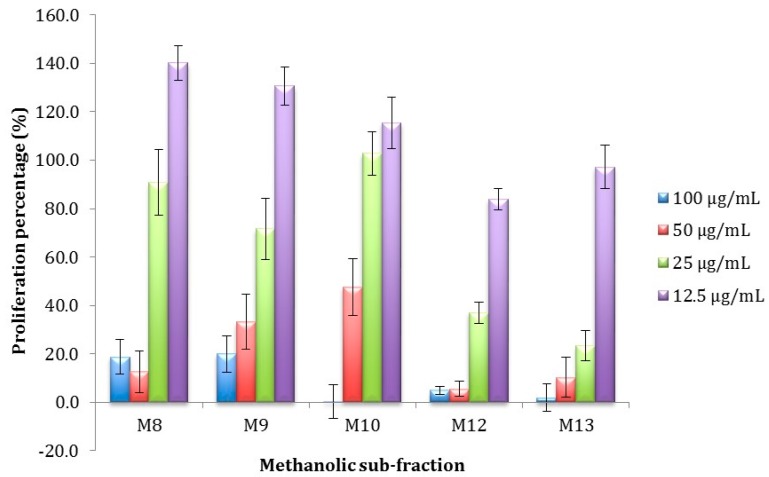
Percentage of proliferation of M12.C3.F6 murine cells exposed to sub-fractions at different lower concentrations. All values represent mean of triplicate determinations ± standard deviation. Control cell cultures were incubated with DMSO (0.5%).

The antiproliferative activity of the five sub-fractions was evaluated at lower concentrations (Figure 3) and their half inhibitory concentration (IC_50_) was calculated. Fractions M12 and M13 showed the highest antiproliferative activity at the lowest concentration with IC_50_ of 19.5 ± 8.6 and 34.9 ± 7.3 for M12 and M13 respectively, this concentrations exhibited significant differences (*p* < 0.05). Fraction M12 was selected for further fractioning since it significantly showed the highest antiproliferative activity.

### 2.3. Fractioning by Open Column Chromatography

In order to continue with the isolation of the bioactive compounds, fraction M12 was subjected to an open column chromatographic procedure. Three fractions were obtained from this chromatographic step and they were coded as M12a, M12b, and M12c. All fractions obtained were analyzed for their chemical structure and antiproliferative activity.

#### 2.3.1. Chemical/Structural Characterization of M12 Fractions

**Figure 4:**
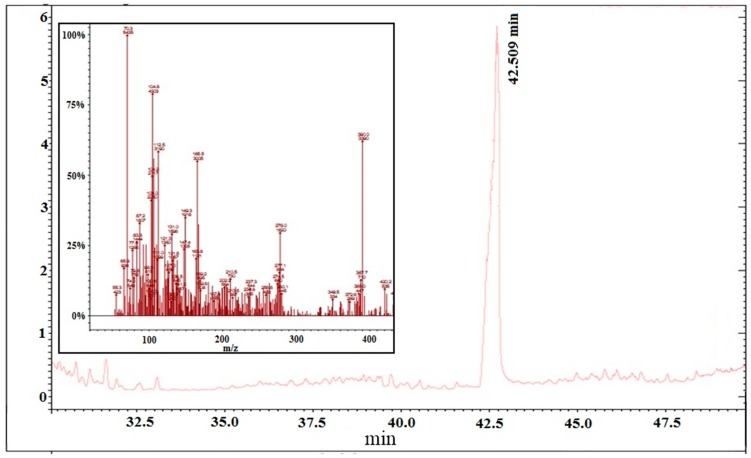
GC-MS spectra of M12a fraction obtained from lipidic extracts of shrimp.

**Figure 5:**
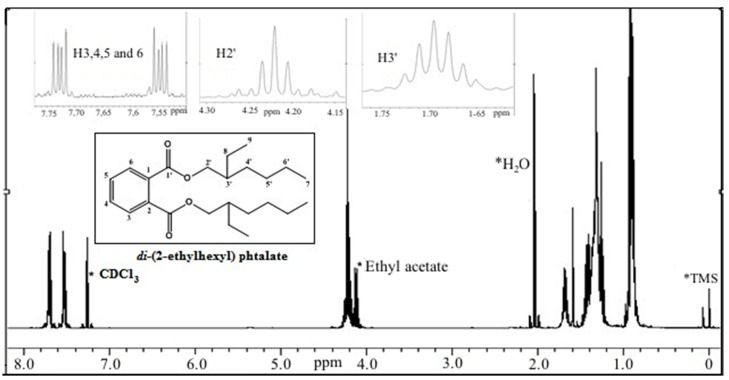
^1^H NMR (in CDCl_3_) spectra of M12a fraction obtained from lipidic extracts from shrimp.

Fraction M12a was obtained as a colorless liquid. GC-MS spectra exhibited a molecular ion peak *m*/*z* = 390, corresponding to the molecular condensed formula C_6_H_4_(C_8_H_17_COO)_2_ of the compound *di*-(2-ethylhexyl) phthalate, as well as the characteristic fragment ions of this compound with *m*/*z* values of 278, 166, 149, and 113 (Figure 4); this data was corroborated by the ^1^H NMR spectra (400 MHz) (Figure 5), where downfield-signals at δ = 7.5–7.75 ppm evidence the presence of hydrogen attached to an aromatic ring in the ortho position. Signals observed at δ = 4.2 and 4.5 ppm may be attributed to protons of adjacent carbons attached to an ester bond (C–O). Finally, chemical shifts at high field (δ = 0.88–1.71 ppm) are attributed to methyl, methylene, and methine protons.

Phthalate derivatives have been previously reported as compounds isolated from various natural sources such as microorganisms [27,28,29], Aloe vera [30], and from marine organisms such as sponges [31] rhizoid [32], and seahorse [33]. The presence of phthalate in several natural sources is consistent with our findings.

Phthalate derivatives have been reported as bioactive compounds, exerting a variety of biological activities such as antioxidant [31,33], antimicrobial [27,29], cytotoxic [27], anti-leukaemic, and antimutagenic [30] potential. Specifically *di*-(2-ethylhexyl) phthalate, has been previously tested as a compound that can induce cell death in mammalian [34] and leukemic [28] cells, and decreasing cell proliferation in human corneal endothelial cells [35].

**Table 2:** Percentage of proliferation of M12.C3.F6 murine cells exposed to methanolic fractions at different concentrations.

Fraction	200 µg/mL	100 µg/mL
M1	129.22 ±16.75 ^a^	113.60 ± 13.22 ^b^
M2	133.53 ± 11.62 ^a^	118.22 ± 8.77 ^b^
M3	129.76 ± 0.51 ^a^	120.17 ± 10.67 ^b^
M4	98.06 ± 3.60 ^b^	117.86 ± 9.05 ^b^
M5	2.004 ± 2.05 ^e^	74.61 ± 5.92 ^c,d^
M6	3.402 ± 3.31 ^e^	69.14 ± 10.94 ^c,d^
M7	57.72 ± 6.02 ^c,d^	171.56 ± 8.20 ^a^
M8	6.379 ± 4.48 ^e^	16.16 ± 4.19 ^e,f^
M9	2.19 ± 0.63 ^e^	3.28 ± 1.26 ^f^
M10	6.20 ± 1.48 ^e^	16.76 ± 4.55 ^f^
M11	13.00 ± 8.505 ^e^	44.83 ± 12.21 ^d,e^
M12	2.67 ± 0.92 ^e^	2.79 ± 2.84 ^f^
M13	8.51 ± 3.79 ^e^	22.84 ± 2.59 ^e,f^
M14	67.63 ± 8.75 ^d^	105.95 ± 13.78 ^b,c^
M15	97.93 ± 5.67 ^b^	136.19 ± 2.69 ^a,b^

All values represent mean of triplicate determinations ± standard deviation. Different letters in a column represent significant differences (*p* < 0.05); Control cell cultures were incubated with DMSO (0.5%) and represent 100% proliferation.

However, anthropogenic contamination of marine environments with phthalates has also been reported [36,37,38], and this kind of compound may be found in both, plant and animal marine species; therefore, the possibility that the antiproliferative phthalate derivative compound isolated from white shrimp in this study might be a product of environmental contamination should be considered.

**Figure 6:**
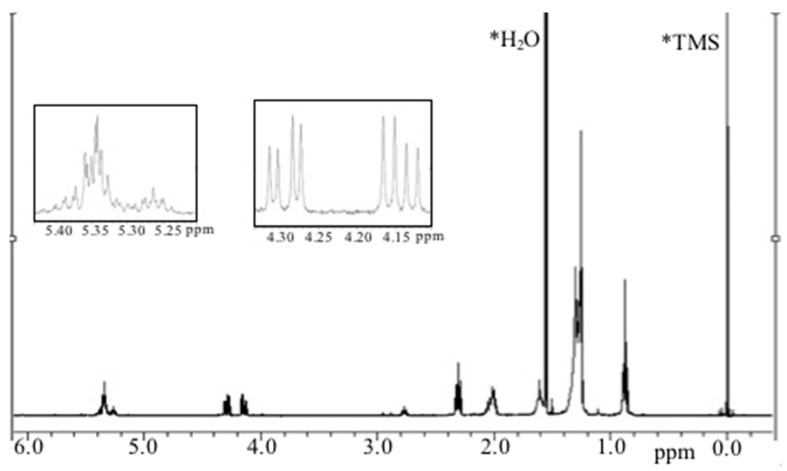
^1^H NMR of M12b lipidic fraction in CDCl_3_.

Regarding fraction M12b, characteristic signals produced by the acyl glycerol type of compound in which glycerol is substituted by unsaturated fatty acids are shown in the ^1^H NMR spectra (400 MHz) (Figure 6). Downfield-signals at δ = 5.3–5.4 ppm suggest the existence of hydrogens that are characteristically attached to carbon atoms participating in a double-bond; signals at δ = 4.1–4.35 ppm suggest protons of an esterified glycerol; up-field signals at δ = 2.7–2.9 ppm are attributed to *bis*-allylic protons of polyunsaturated fatty acids; however, the characteristic signal of docosahexaenoic acid (DHA) (δ = 2.4 ppm) was not present in the spectra, suggesting that proton signals could be attributed to the presence of eicosapentaenoic acid (EPA) [39]. The other signals may be attributed to oleic acid; both fatty acids have been reported previously as two of the main fatty acids along with DHA and linoleic acid [40].

**Figure 7:**
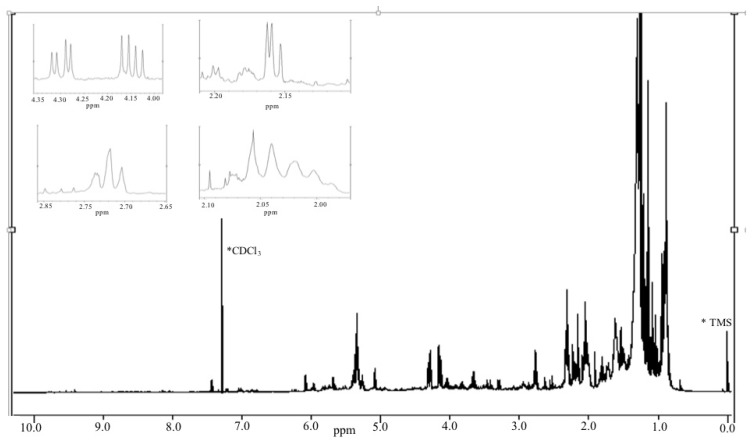
^1^H NMR of M12c lipidic fraction in CDCl_3_

The ^1^H NMR spectra (400 MHz) (Figure 7) of fraction M12c also showed characteristic signals of those produced by acyl glycerol type of compounds, in which glycerol is substituted by saturated and polyunsaturated fatty acids. Downfield-signals at δ = 5.3–5.4 ppm, evidence of the existence hydrogen that are characteristically attached to carbon atoms participating in a double-bond, signals at δ = 4.1–4.35 ppm are evidence of protons of an esterified glycerol, up-field, signals at δ = 2.7–2.9 ppm are attributed to *bis*-allylic protons of polyunsaturated fatty acids, and other signals are characteristic of saturated fatty acids such as stearic and palmitic acids.

#### 2.3.2. Antiproliferative Activity of M12 Fractions

**Figure 8:**
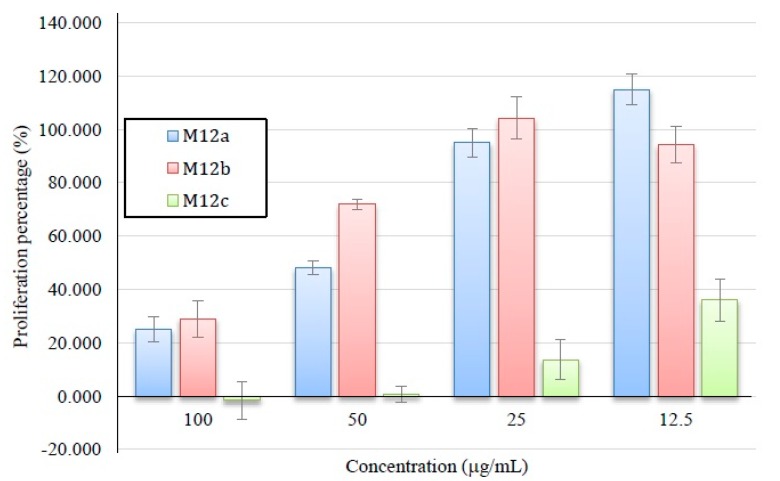
Antiproliferative activity of M12 sub-fractions. All values represent mean of triplicate determinations ± standard deviation. Control cell cultures were incubated with DMSO (0.5%), which represented 100% proliferation.

M12 sub-fractions were further tested in M12.C3.F6 cells with concentrations from 12.5 to 100 µg/mL in order to determine which of the compounds exerted the highest activity (Figure 8). M12c, had the highest activity with an IC_50_ further estimated of 11.33 µg/mL.

Even though previous studies have reported the presence of bioactive compounds in shrimp, most of these were not extracted from shrimp muscle. Antioxidant activity was detected in extracts obtained from several shrimp byproducts such as head [41,42] and shell [43]. Anti-inflammatory properties have been reported in shrimp shell [43] and antimutagenic activity was detected in shrimp muscle using a crude extract [44]. In all these reports, the bioactivities have been attributed to compounds of a carotenoid nature. Nevertheless, only crude extracts were studied and the absorbance at visible spectra wavelength reported, attributing the biological activity to carotenoids; the extracts were not purified in order to identify the chemical structure of the compound responsible for the activity. In our laboratory, we have reported the presence of antiproliferative compounds, initially extracted from shrimp muscle lipidic fraction, which were obtained from fractions after a series of thin-layer chromatography procedures [45]. After an isolation procedure involving solvent partitioning, open-column chromatography and semi-preparative RP-HPLC, the present research work provides evidence that the compound that exerts the highest antiproliferative activity in shrimp are triglycerides esterified with polyunsaturated fatty acids, compounds that have previously been reported as biologically active [46,47].

## 3. Experimental Section

### 3.1. Testing Species

Shrimp (*Litopenaeus vannamei*) was obtained from a local market in Hermosillo, Sonora, Mexico, and transported in ice to the University of Sonora Seafood Laboratory. Edible portions of shrimp were separated, packed, and stored at −20 °C until analysis. The extraction of the shrimp lipidic fraction was carried out according to the methodology reported by [48]. A 100 g shrimp muscle sample was homogenized with five parts of CHCl_3_ (*w*/*w*) in a blender at high speed for 1 min and the resulting mixture was poured into an Erlenmeyer flask and agitated for 40 min with the aid of a Wrist Action Burrel Shaker (Burrel Corporation, Pittsburg, PA, USA). The mixture was filtered through a Whatman No. 1 filter paper (Whatman, Clifton, NJ, USA) under vacuum and the filtrate was evaporated to dryness under reduced pressure at 40 °C. The lipidic extract was then re-dissolved in methanol-hexane (1:1 *v*/*v*), agitated for 30 min and filtrated again through a Whatman No. 1 filter paper under vacuum. The immiscible phases were separated in a separating funnel and concentrated under reduced pressure at 40 °C, re-dissolved in chloroform, and dried under N_2_ stream. All the process was performed in darkness.

### 3.2. Analysis of Lipidic Composition by RP-HPLC

Fractionation of lipidic fractions was carried out by semi-preparative HPLC using an Agilent Technologies HPLC station (Palo Alto, CA, USA) equipped with a Zorbax Eclipse XDB-C18 semi-preparative column (250 mm ° 9.4 mm internal diameter; 5 µm particle size; Agilent Technologies). A guard column made of the same material was also used. Aliquots of 100 μL from each extract were injected into the column according to the modified procedure of [49]. Elution of components was performed using a flow rate of 2 mL/min and was continuously monitored by diode array detector (DAD) (Agilent Technologies) at 450 nm. Column temperature was maintained at 20 °C. Solvents used for elution were water (A), acetone (B), and hexane (C). Lipids were eluted from the column using a linear gradient from 70% A, 30% B to 100% B in 5 min, and then a linear gradient from 100% B to 70% B, 30% C up to minute 20 with a 3-min re-equilibration period at the initial conditions before application of the next sample. Fractions were collected using an Agilent Technologies fraction collector with a flow delay of 30 s, for further chemical characterization. The collected fractions were individually tested for antiproliferative activity.

### 3.3. Isolation of Bioactive Component by Column Chromatography

**Figure 9:**
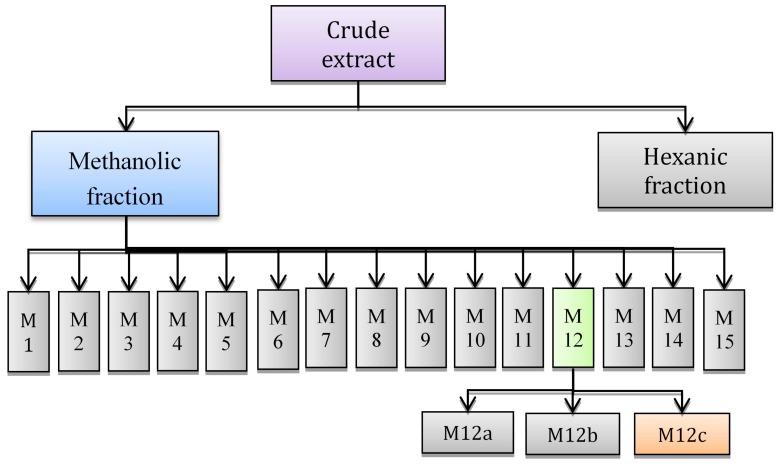
Schematic for separation and isolation of antiproliferative fractions from shrimp.

The sub-fraction obtained by HPLC with the highest antiproliferative activity was subjected to open column chromatography under gravity on silica gel (3.5 cm × 20 cm using silica, 60–120 mesh, Sigma, St. Louis, MO, USA). Fraction M12 was poured onto the column and eluted using a mobile phase consisting of hexane:ethyl acetate (99:1). The eluents were monitored using TLC (Thin Layer Chromatography) testing plates coated with silica gel and contents were revealed using an iodide solution and observed under *UV* light. The fractions containing similar signals were combined. Schematic representation of the isolation procedure is showed in Figure 9.

### 3.4. Cell Lines

Cell line M12.C3.F6 (murine B-cell lymphoma) was kindly provided by Dr. Emil R. Unanue (Department of Pathology and Immunology, Washington University at St. Louis, MO, USA). Cell cultures were cultured in Dulbecco’s modified Eagle’s medium (DMEM) (Gibco, Grand Island, NY, USA) supplemented with 5% heat inactivated fetal calf serum and grown at 37 °C in an atmosphere of 5% CO_2_.

### 3.5. Antiproliferation Assay

The effect of shrimp lipidic fractions on the proliferation of the M12.C3F6 cell line was determined using the standard MTT assay [50]. Briefly, 10,000 cells (50 μL) were placed in each well of a flat 96-well plate. After 12 h incubation at 37 °C in an atmosphere of 5% CO_2_ to allow cell attachment, cell cultures were incubated with 50 μL of medium containing various concentrations of the lipidic fractions and incubated for 48 h. Shrimp lipidic fractions were first re-suspended in DMSO and diluted in supplemented DMEM media. Control cell cultures were incubated with DMSO (final concentrations of DMSO 0.06%–0.5% *v*/*v*). Control cell cultures did not show any evidence of cell damage. Prior to the last 4 h of the cell culture, 10 μL of MTT stock solution (5 mg/mL) were added to each well. Formazan crystals formed were dissolved with acidic isopropanol and the plates were read in an ELISA plate reader (Benchmark Microplate Reader; Bio-Rad, Hercules, CA, USA) using a test wavelength of 570 nm and a reference wavelength of 630 nm. Plates were normally read within 15 min after the addition of isopropanol.

### 3.6. ^1^H NMR Analysis

Measurements were performed using an Agilent Technologies equipment operating at 400 MHz. Each fraction was dissolved in CDCl_3_ (500 µL; Sigma-Aldrich, Saint Louis, MI, USA) with a small amount of tetramethylsilane (TMS) as internal standard and the resulting mixture was placed into a 5 mm diameter ultra-precision NMR sample tubes. Chemical shifts were recorded in ppm, using TMS proton signal as an internal standard.

### 3.7. GC-MS Analysis

GC-MS analysis was performed using a Varian 450 chromatograph equipped with a VF-5ms column (30 m × 0.25 mm internal diameter; 0.25 µm film). Aliquots of 3 μL were injected into the column; elution of components was performed using a flow rate of 1 mL/min. Compounds were detected using a Varian 220 mass detector (50–500 *m*/*z*).

### 3.8. Statistical Analysis

Data were analyzed using analysis of variance (ANOVA) with Tukey-Kramer and Duncan’s multiple comparison tests (Number Cruncher Statistical Software (NCSS), Kaysville, UT, USA).

## 4. Conclusions

The lipidic extract of white shrimp muscle is a source of chemopreventive compounds, and even though the biological activity of shrimp has been previously attributed to the presence of carotenoid compounds, mainly astaxanthin, this study demonstrates that the compounds mainly responsible for the antiproliferative activity are triglycerides substituted with polyunsaturated fatty acids, eicosapentaenoic and saturated fatty acids. While these bioactive triglycerides have shown promising antiproliferative properties against murine tumorous cell lines, further research work is necessary to assess their actual chemotherapeutic potential.
